# Mortality and life expectancy of professional fire fighters in Hamburg, Germany: a cohort study 1950 – 2000

**DOI:** 10.1186/1476-069X-5-27

**Published:** 2006-10-04

**Authors:** Norbert L Wagner, Jürgen Berger, Dieter Flesch-Janys, Peter Koch, Anja Köchel, Michel Peschke, Trude Ossenbach

**Affiliations:** 1 Department of Environmental Health Engineering, Sri Ramachandra Medical College & Research Institute 1, Ramachandra Nagar, Porur, Chennai 600 116, India; 2 Epidemiology Working Group, Department of Science and Health and Institute for Medical Biometrics and Epidemiology, Winterhuder Weg 29, 22085 Hamburg, Germany; 3 Occupational Medical Service – Personnel Department, Free and Hanseatic City of Hamburg Alter Steinweg 4, 20459 Hamburg, Germany

## Abstract

**Background:**

The healthy worker effect may hide adverse health effects in hazardous jobs, especially those where physical fitness is required. Fire fighters may serve as a good example because they sometimes are severely exposed to hazardous substances while on the other hand their physical fitness and their strong health surveillance by far exceeds that of comparable persons from the general population.

**Methods:**

To study this effect a historic cohort study was conducted to assess mortality and life expectancy of professional fire fighters of the City of Hamburg, Germany. Fire departments and trade unions questioned the validity of existing studies from outside Germany because of specific differences in the professional career. No mortality study had been conducted so far in Germany and only few in Europe. Information on all active and retired fire fighters was extracted from personnel records. To assure completeness of data the cohort was restricted to all fire fighters being active on January 1, 1950 or later. Follow up of the cohort ended on June 30th 2000. Vital status was assessed by personnel records, pension fund records and the German residence registries. Mortality of fire fighters was compared to mortality of the Hamburg and German male population by means of standardized mortality ratios. Life expectancy was calculated using life table analysis. Multivariate proportional hazard models were used to assess the effect of seniority, time from first employment, and other occupational characteristics on mortality.

**Results:**

The cohort consists of 4640 fire fighters accumulating 111796 person years. Vital status could be determined for 98.2% of the cohort. By the end of follow up 1052 person were deceased. Standardized Mortality Ratio (SMR) for the total cohort was 0.79 (95% CI, 0.74–0.84) compared to Hamburg reference data and 0.78 (95% CI, 0.74–0.83) compared to National German reference data. Conditional life expectancy of a 30 year old fire fighter was 45.3 years as compared to 42.9 year of a German male in normal population. Job tasks, rank status and early retirement negatively influenced mortality. For fire fighters with comparably short duration of employment the mortality advantage diminished with longer time since first employment. SMR of persons who retired early was 1.25 (95% CI, 1.13–1.60) in reference to the general German population and the SMR of 1.71 (1.18–2.50) in the multivariate regression model.

**Conclusion:**

A strong healthy worker effect was observed for the cohort, which diminished with longer time since first employment for fire fighters with shorter duration of employment, as expected. The negative effects on mortality of job tasks, rank status and in particular early retirement indicate the presence of undetermined and specific risks related to occupational hazards of fire fighters.

## Background

At the end of the 1990es a raise of retirement age for police and fire fighters was discussed in the German parliament in the context of reforms of social welfare system, pension fund financing. The retirement age of 60 years was supposed to be raised to 62 years. Trade unions and fire departments opposed these plans arguing that life expectancy of fire fighters was already dramatically shorter compared to general population. Arbitrarily collected and published data from fire departments indicated a low average age at death [1]. Later inquiries showed that these self-collected data were wrong.

All previously published mortality data from other countries (Table 1) indicated a lower mortality with standardized mortality ratios between 0.76 and 0.99. However, in the light of the self-collected data in Germany these studies were considered unreliable and "not applicable" by both, the fire departments and the trade unions.

**Table 1:** Published studies on mortality of fire fighters

**Author**	**Year**	**City/Country**	**Size of Cohort**	**Period of recruitment**	**End of Observation**	**SMR**	**95% CI**
Musk [30]	1978	Boston/USA	5655	1915–1975	7/1975	0.91	na
Eliopulos [31]	1984	Western Australian Fire Brigade	990	10/1939 – 12/1978	12/1978	0.80	0.67–0.96
Vena [32]	1987	Buffalo/USA	1867	1950 – 1979	1979	0.95	na
Heyer [33]	1990	Seattle/USA	2289	1/1945 – 12/1979	12/1983	0.76	0.69–0.85
Hansen [34]	1990	Denmark Census 1970	886	1970	1980	0.99	0.75–1.29
Beaumont [35]	1991	San Francisco/USA	3066	1940 – 1970	1982	0.90	0.85–0.95
Demers [9]	1992	Seattle. Tacomo. Portland/USA	4546	1944 – 1979	1989	0.81	0.77–0.86
Guidotti [11]	1993	Alberta/Canada	3328	1927 – 1987	1987	0.96	0.87–1.07
Aronson [36]	1994	Toronto/Canada	5995	1950 – 1989	1989	0.95	0.88–1.02
Tornling [37]	1994	Stockholm/Sweden	1116	1931 – 1983	1986	0.82	0.72–0.91
Deschamps [38]	1995	Paris/France	830	1973 – 1991	1991	0.52	0.35–0.75
Baris [39]	2001	Philadelphia/USA	7789	1925 – 1986		0.96	
Present study	2001	Hamburg/Germany	4557	1.1.1950-30.6.2000	6/2000	0.78	0.74–0.83

na: not available

In July 2000 the head of fire department of the Free and Hanseatic City of Hamburg requested the Occupational Medical Service to conduct a scientific study to answer to this question. The validity of existing studies from outside Germany was questioned by fire departments because of presumed specific differences in the professional career in Germany like frequent life long employment, work till later age, remaining a fire fighters longer because of specific German pension benefits. No mortality study had been conducted so far in Germany and very few in Europe. In collaboration with the Institute of Mathematics and Computational Sciences in Medicine of the School of Medicine at University of Hamburg a retrospective cohort study was initiated in autumn 2000.

This study reports the findings of a retrospective cohort study on the mortality of fire fighters of the Fire Department of the City of Hamburg, Germany. The objectives were to establish the life expectancy and standardized mortality ratios compared to the Hamburg and the national reference population. We put special emphasis on disentangling the suspected strong healthy worker effect in this cohort from the effects of potential chemical exposures and heavy work load.

## Methods

All male fire fighters of the Fire Department of the City of Hamburg, Germany being active between January 1, 1950 and June 30, 2000 (end of follow-up) were included in the study population. They were all full-time, professional employees. The number of female fire fighters was negligible and thus they were excluded. We included only active fire fighters and no administrative personnel of the Fire Department.

Date of birth, date of death, date of employment, date of end of active duty, reason of end of active duty, duration of employment according to the type of tasks (fire fighting & rescue service vs. administration) and rank were obtained for pensioners and active fire fighters from the Hamburg Fire Department. This included e.g. card files of pensioners and the archive of personnel records.

Initially we identified a total of 4805 fire fighters who had ever worked in the Fire Department of the City of Hamburg. To ensure completeness of data sets, the cohort and due to the availability of reference mortality data we restricted analysis to fire fighters who were active on January 1, 1950 or were employed before the end of follow up on June, 30, 2000. Thus 141 persons (including 125 deceased) were not included in the cohort because they had left the department before January 1, 1950. In addition, we excluded 10 cases with missing dates of entry, 2 cases with age at employment lower than 15 years and 12 pensioners with missing dates of leaving the fire department.

The database from the personnel department was crosschecked with other data sources. By these additional data sources we added 122 persons out of the files of retired fire fighters, 149 persons out of the personnel archive paper files. We corrected 22 dates of death with databases from the retirement and inheritance fund.

Comparing the database to other publications corroborates the presumption of almost 100% completeness: the department yearbook of 1972 lists 1026 persons for 1955 compared to our 1021, for 1967: 1660 vs. 1657, for 1972: 1604 and 1625 vs. 1644.

Vital status was determined from the department's personnel records and pension fund. No information regarding causes of death was available in these data sources and no additional funding was available to ascertain this information from the death certificate registries. For 381 persons vital status remained unknown. We obtained further vital status information from the German municipal residents' registration and vital status offices. These mandatory registries capture residence and vital status of persons who e.g. moved to another city. Vital status for 77% of these 381 persons (227 living, 66 dead) was obtained. Unclear vital status and hence lost to follow-up were 88 persons.

The final cohort entering the statistical analysis thus included 4557 fire fighters with known vital status.

Standardized mortality ratios (SMR) were calculated using the Hamburg mortality rates from the Department of Statistics of the City of Hamburg and German reference rates provided by the German Cancer Research Centre, Heidelberg. SMR computations used the software SMRPER [2]. Confidence intervals were calculated assuming a Poisson distribution for the observed cases. We examined mortality in stratified analyses by year of entry, type of task, rank group (rank), duration of employment and time since first employment (duration of observation).

Life expectancy for different ages at entry into the fire department were calculated by life table analysis according to the Manual of Mortality Analysis [3] using the time period and age specific death rates of the cohort. Comparable life expectancies for the Hamburg and German population were computed from weighted death rates of Hamburg and Germany. Weights were computed by age- and date-of birth-specific numbers of fire fighters.

Multivariate subgroup analyses were conducted with time dependent proportional hazard models using rank group, age at follow up, year of first and duration of employment as predictors. The analyses were performed with SPSS. For the purpose of analysing work time-related effects, only persons born January 1, 1950 or later were included.

Details which could have determined the exposure better besides the classification into "fire fighting & rescue" vs. "administrative" groups were not available. Further classification for a more elaborate job-exposure matrix (for example: position in the team while fire fighting) was considered inappropriate and not reliable. The reasons for this decision were:

a) Professional fire fighters in Germany rotate almost on weekly – if not daily – base into different positions in the team.

b) Major fires with very high exposures are rare. In Hamburg major structural and industrial fires occur with a sequence of one to two per year for each fire fighter.

c) High exposures (for example, from carcinogenic chemicals such as diesel fumes) occur during maintenance and routine work in the station.

d) Even when major fires occur it is unclear who in the brigade has actually the highest exposure. The attack team have also the highest level of personal protection (SCBA); two attack teams (on ground and on ladder) might have completely different exposure levels; the commander who tries to get a good glimpse on the situation but doesn't use his mask in order to be able to communicate better via walkie-talkie might even have the higher exposure to burn products and fumes. It is known that the highest exposures are often during overhaul and destruction, so the position of the fire fighter during the actual fire is not predictive of his/her exposure during the entire course of the sortie.

e) Even if a position or task could have been documented the use of SCBA is unknown.

## Results

The final cohort entering the statistical analysis thus comprised 4557 persons: 2169 (47.6%) active fire fighters and 2388 (52.4%) pensioners. During follow up 1052 deaths were observed.

Table 2 shows the descriptive statistics for the cohort. Half of the included persons were employed before 1966 and half left the fire brigade before 1985. Half of the workers have a maximum duration of employment of 18 years.

**Table 2:** Descriptive statistics for the fire fighter cohort

**Variable**	**Median**	**Minimum**	**Maximum**
Date of birth	1942	1885	1980
Date on entry	1966	1950	2000
Date of leaving	1985	1950	2000
Date of death	1981	1950	2000
Age at employment	26	15	65
Age at end of observation	55	20	103
Duration of employment	18	0.02	47

### Reasons for leaving active duty

Regular retirement was the reason for 1419 fire fighters (59.4%) and early retirement for 470 (19.7%). A total of 237 fire fighters (9.9%) asked to leave, 214 fire fighters (9.0%) died before retirement. For 48 fire fighters (2.0%) we could not determine their reasons to leave active duty in the department.

### Life expectancy

Life expectancies for fire fighters in comparison to male persons from the German and Hamburg population according to different ages at employment are shown in Table 3. The life expectancy of a fire fighter aged 20–24 year at entry was 54.9 years, 2.7 and 2.8 years more in comparison to the German or Hamburg reference respectively. For a fire fighter aged 30–34 the advantage in life expectancy was 2.2 years compared to Hamburg and 2.4 years compared to Germany.

**Table 3:** Age specific life expectancies for fire fighters in comparison to Hamburg and Germany reference population

**Age Group**	**Fire Fighters Cohort Hamburg**	**Germany Reference**	**Difference to German Reference**	**Hamburg Reference**	**Difference to Hamburg Reference**
20–24	54.9	52.2	2.7	52.1	2.8
25–29	50.2	47.6	2.6	47.8	2.4
30–34	45.3	42.9	2.4	43	2.2
35–39	40.4	38.2	2.2	38.4	2.1
40–44	35.7	33.5	2.2	33.7	2
45–49	31.1	29	2.1	29.3	1.8
50–54	26.7	24.7	1.9	25	1.6
55–59	22.5	20.6	1.8	21	1.5
60–64	18.4	16.8	1.6	17.3	1.2
65–69	14.4	13.4	1	13.8	0.6
70–74	11	10.4	0.7	10.8	0.3
75–79	8.4	7.8	0.6	8.2	0.2
80–84	6.4	5.7	0.7	6	0.3
85–89	4.7	3.9	0.9	4.1	0.6

### Standardized Mortality Ratios

Overall SMR using the age specific death rates of Hamburg and Germany were notably reduced (Table 4). SMR for the cohort was 0.79 with a 95% CI of 0.74–0.84 (Reference Hamburg) and 0.78 with a 95% CI of 0.74–0.83 (Reference Germany)). Because the results of the SMR computation were similar for both reference populations the subgroup analysis given below only refers to the German population.

**Table 4:** Standardized mortality ratios compared to Hamburg and German Reference Population

	***N***	***Observed***	***Expected***	***Person years***	***SMR***	***95% CI***
**Reference Hamburg**	4557	1052	1331	111795.7	0.79	0.74–0.84
**Reference Germany**	4557	1052	1345.3	111795.7	0.78	0.74–0.83

Stratification by year of entry shows a reduction in mortality over time. For persons who were already active on the 1/1/1950 the SMR was 0.85 (95% CI, 0.79–0.91). For fire fighters who were employed between 1950 and 1954 the SMR was 0.66 (95% CI, 0.49–0.88), decreasing to 0.47 (95% CI, 0.31–0.68) for the last stratum (entry 1970–2000). A further stratification of year of entry by type of duties (fire fighting & rescue service) yielded a similar trend in the SMRs (data not shown).

More detailed analysis on SMR in sub-groups is given in Table 5. All time- and age-related variables were analysed independently. Multivariate analysis is discussed below.

**Table 5:** Standardized mortality ratios by risk factors

	***N***	***Observed***	***Expected***	***Person years***	***SMR***	***95% CI***
**Year of employment**	4557					
Employed on 1. Jan 1950	981	802	943.7	31702.2	0.85	0.79–0.91
1950–1954	125	47	71.5	5334.6	0.66	0.49–0.88
1955–1959	408	82	122	16300.8	0.67	0.54–0.83
1960–1964	384	50	74.8	13926.4	0.67	0.50–0.88
1965–1969	428	43	73.7	14109.8	0.58	0.42–0.79
1970–2000	2231	28	59.6	30421.9	0.47	0.31–0.68
**Predominant type of tasks**	4470					
Fire fighting and rescue service	4116	972	1237.2	104685.4	0.79	0.74–0.84
Administrative jobs as fire fighter	354	26	49	5087.8	0.53	0.35–0.78
**Rank groups**	4309					
Middle ranks [see note]	3631	900	1101	89142.8	0.82	0.77–0.87
High ranks	632	72	150.2	18172.4	0.48	0.38–0.60
Higher ranks	46	14	17.3	1243.9	0.81	0.44–1.36
Combined: high and higher ranks	678	86	167.5	19416.3	0.51	0.41–0.63
**Reasons for leaving**	2124					
Own request	236	33	29.5	4033.1	1.12	0.77–1.57
Early retirement	469	131	96.9	4989.6	1.35	1.13–1.60
Regular retirement	1419	644	816.2	16409.8	0.79	0.73–0.85

Note: Reference for the calculations is the German general population. The "middle rank" is actually the lowest group a fire fighter can join.

Mortality declines the later the year of employment. SMR changes from 0.85 (95% CI, 0.79–0.91) for persons employed at 1/1/1950 to 0.47 (95% CI 0.31–0.68) for fire fighters who joined between 1970 and 2000 (Table 5).

For persons with available data for the type of tasks (N = 4470) differences in mortality were observed: fire fighters who spent more than 50% of their working time in the fire fighting & rescue service showed a higher SMR (0.79; 95% CI, 0.74–0.84) than fire fighters with more than 50% of their working time in the administration (SMR, 0.53; 95% CI, 0.35–0.78). Categorizing the cohort in two subgroups who had worked exclusively in the fire fighting & rescue service or in the administration as fire fighters yielded SMRs of 0.81 (95% CI, 0.75–0.87) and 0.43 (95% CI, 0.12–1.10), respectively (data not shown).

Dividing the cohort into 'rank groups' according to the German state employment categories (in German: "mittlerer, gehobener und höherer Dienst") as indicators of socio-economic status showed higher mortality (SMR, 0.82; 95% CI, 0.77–0.87) for the 'middle rank' than for fire fighters in the 'high' or 'higher rank' group combined (SMR, 0.51; 95% CI, 0.41–0.63) with 4309 available data sets (Table 5). The 'middle rank' is the lowest income group a fire fighter can join in the last decades. The terminology has historical reasons: the "low rank" category was abandoned in the middle of last century. Depending on prior qualification fire fighters can join the higher rank groups directly. For 248 persons reliable information on rank was not available.

Regarding the 'reason of retirement' persons with a regular retirement showed a reduction in mortality (SMR, 0.79; 95% CI, 0.73–0.85) compared to the German reference population (Table 5). Fire fighters who left the department because of other reasons showed an increase in mortality: early pensioners (SMR, 1.35; 95% CI, 1.13–1.60) left the department for health reasons and other fire fighters who left the fire department on their own request (SMR, 1.12; 95% CI, 0.77–1.57) for instance to move to a different city.

Table 6 shows the result of the SMR calculations for duration of and time since first employment simultaneously. The SMR in the first five years after joining the fire fighters was only 0.3 (0.12–0.61). This decreased mortality seemed to disappear for the subgroups of fire fighters with a total duration of employment of less than 10 years, however, numbers in these subgroups were quite small. For the subgroups with longer duration of employment the mortality appeared to increase with longer period of follow up, but after 30 years of observation the SMR continued to be significantly below 1.

**Table 6:** SMRs, by duration of employment and time since first employment

***Duration of observation***	***0–4 years***	***5–9 years***	***10–19 years***	***20–29 years***	***30+ years***	***Total***
						
***Duration of employment***						
***0–4 years***	0.3 (7/23.6) 0.12–0.61	4.76 (4/0.8) 1.30–12.19	0.5 (1/1.9) 0.01–2.93	1.24 (4/3.2) 0.34–3.20	1.66 (10/6.0) 0.80–3.07	0.73 (26/35.5) 0.80–1.07
***5–9 years***		0.51 (12/23.7) 0.26–0.88	1.04 (3/2.9) 0.21–3.04	0.59 (2/3.39) 0.07–2.13	3.62 (4/1.1) 0.99–9.31	0.68 (21/31.1) 0.42–1.03
***10–19 years***			0.60 (37/61.6) 0.42–0.83	1.5 (11/7.3) 0.75–2.70	0.76 (9/11.8) 0.35–1.45	0.71 (57/80.7) 0.53–0.92
***20–29 years***				0.60 (55/91.5) 0.45–0.78	0.67 (20/29.9) 0.41–1.03	0.62 (75/121.4) 0.49–0.77
***30+ years***					0.55 (71/129.5) 0.43–0.69	0.55 (71/129.5) 0.43–0.69
***Total***	0.3 (7/23.6) 0.12–0.61	0.65 (16/24.54) 0.37–1.06	0.62 (41/66.38) 0.44–0.84	0.68 (72/105.4) 0.53–0.86	0.64 (114/178.3) 0.53–0.77	0.63 (250/398.2) 0.55–0.71

Note: fire fighter cohort employed after 1/1/1950 (SMR, # of observed/expected cases, 95% CI)

### Multivariate subgroup analysis with proportional hazard models

To include time dependent covariates a subgroup was formed excluding all workers already active on the 1/1/1950. Table 7 presents the results of the proportional hazard model (N = 3576) using following time dependent covariates: duration of employment, year of employment, age at employment, rank group, reason for leaving simultaneously. Adding the variable 'type of task' into the model did not improve it. It was hence not included.

**Table 7:** Multivariate Subgroup Analysis

**Covariates**	**Relative risk (95%CI)**
**Rank group** (reference "middle rank")	
High and higher rank	0.39 (0.24–0.62)
**Age at employment** (reference 15–25 years)	
25–29 years	0.91 (0.66–1.26)
> = 30 years	2.38 (1.36–4.15)
**Year of employment** (reference 1950–54)	
1955–1959	0.98 (0.56–1.71)
1960–1964	0.77 (0.37–1.58)
1965–1969	0.57 (0.25–1.27)
1970–2000	0.46 (0.14–1.57)
**Duration of employment** (reference 0–5 years)	
5–<10 years	2.43 (0.93–6.34)
10–<20 years	0.89 (0.46–1.74)
20–<30 years	0.35 (0.20–0.62)
> = 30 years	0.42 (0.23–0.75)
**Early retirement** (reference all others)	
Early retirees	1.71 (1.18–2.50)

#### Duration of employment

A longer duration of employment (more than 20 years) in comparison to the reference group '0–5 years duration of employment' is associated with a lower mortality risk (20–30 years RR, 0.35; 95% CI, 0.20–0.62; and > = 30 years RR, 0.42; 95% CI, 0.23–0.75). A duration of employment of 5–10 years seems to be connected with increased risk (RR, 2.43 95% CI, 0.93–6.34) but it includes a large statistical uncertainty. We also computed the effect of duration of employment as a continuous covariate in this model and found a RR of 0.73 (95% CI, 0.61–0.87) for each 10 years of employment (result not shown in Table 7).

#### Year of entry

A later date of entry is associated with a lower mortality risk. In comparison to the reference group 'entry between 1950–1954' the mortality risk for the group 'entry after 1970' is reduced to 46 % (RR, 0.46; 95% CI, 0.14–1.57).

#### Age at entry

The age of entry had been categorised in 3 groups. In comparison to the reference group 'age 15–25 years' the mortality of the group 25–30 years reveals no difference. An increased relative risk has been observed for the workers with an age at entry higher than 30 years (RR, 2.38; 95% CI, 1.36–4.15).

#### Rank group

A lower risk was found for the group of the high and highest rank group combined in comparison to the middle rank group (RR, 0.39; 95% CI, 0.24–0.62).

#### Early retirement

Workers who leave the fire department due to early retirement have an increased relative risk (RR, 1.71; 95% CI, 1.18–2.50) in comparison to all others.

## Discussion

This paper reports on the first cohort study undertaken in Germany to examine the mortality of fire fighters with its typical, quasi lifelong employment of professional fire fighters. We are confident that the cohort of fire fighters is complete for the time period after 1950. It constitutes the largest cohort study of fire fighters' mortality in a European country.

The study was limited by the fact that we could not collect a detailed exposure history of fire fighters and data on causes of death.

Taking runs as a proxy parameter for exposure is not established as standard in the research on fire fighter risks. The differences in job tasks, wind direction or protective equipment on jobs at the same fire do not allow taking number of runs as proxy. The exposure matrix of fire fighters is highly complex as time at fire does not indicate that for example protective equipment was worn or not [28].

Neither the evaluation of causes of deaths nor the inclusion a control group such as officers from the Hamburg Police Department was funded by the Fire Department. We tried to account for this limitation by using the Cox regression model with internal comparison groups to make the effects of different risk factors inherent to the occupation visible.

### Overall mortality

Our results show that the mortality of the fire fighter cohort of Hamburg is about 20% lower than the mortality of the reference population. They confirm findings from most of the other published studies from different countries (Table 1). However, our findings are in the lower range of previously published studies. It might indicate that the selection processes and the intensive medical surveillance programs in Germany have a greater effect than the programs in other countries with lower standards (see discussion below).

The lower mortality of fire fighters in comparison to the general population is probably influenced by the healthy worker effect in several aspects. The question how much the healthy worker effect masks a potentially negative effect of occupation on mortality arises in all occupational cohort mortality studies [4,5]. The reference to 'general population' is convenient and – as it was the case here – often the only financially feasible way. Unfortunately it is not the best comparison group to determine the occupation-induced mortality risks because of the selection of cohort members based on health status and risk factors at the beginning of work.

Using definitions according to Choi [6], several components of the healthy worker effect (HWE), e.g. the healthy hired, low-risk hired, worker healthier and the healthy worker survivor effect probably led the observed low mortality. In general the magnitude of the healthy worker effect is estimated to be around 20% advantage in mortality (see [4] for further discussion). Our result (SMR of 0.78) is very similar to that.

We observed a decline of the healthy worker effect with increasing time since first employment. This effect was more pronounced for the subgroups with a total duration of employment of less than ten years. This observation is consistent with the assumption that the "healthy hired" component disappears within this time frame from date of first employment. For fire fighters with longer duration of employment (10–29 years) the mortality advantage is also declining, but the SMR does not increase to 1. This may probably reflect a levelling off of the "low-risk-hiring" component. Finally, the SMR for the subgroup with working time durations of more than 30 years was 0.55 (95% CI, 0.43–0.69), i.e. lower than those for the other subgroups with shorter duration but the same time since first employment. This indicates a pronounced long term effect of the "work healthier" and the "healthy survivor" component of the healthy worker effect.

Physical and medical fitness for professional and voluntary fire fighters is required nationwide in Germany using common standards. This constitutes a major difference to the US American system where regular physical performance tests are suggested by National Fire Protection Association (NFPA)/USA [7] but not regularly required on national level [8]. However, on state and/or community level in the USA regular physical performance tests are sometimes mandatory.

The stringent selection process in Germany demands physical and psychological health and fitness. After joining there are regular and intensive medical examinations: until the age of 50 every 3 years, beyond 50 every year. These tests include stress-ECG to evaluate standardized physical fitness and a fitness test with heavy respiratory protection gear on the obstacle course. In the stress-ECG fire fighters below 30 years of age have to perform at an energy level of three Watt per kilogram of bodyweight. Fire fighters above 30 years of age have to show the same level of performance reduced by one percent per year of age above 30 years. The capacity to perform at an energy level of 200 Watt minimum has to be proven at all occasions. In addition to the fitness test, other medical criteria for vision test, audiometry, lung function test and acceptable blood pressure response and heart rates at stress-ECG have to be met.

The clearance to wear protective gear i.e. the permission to serve in the brigade is cancelled once the fitness tests are failed. Also fire fighters in mostly administrative duty keep themselves fit and pass the medical endurance tests as they might have to go out on the scene in major emergencies. Only these were included in the comparison between 'fire fighting' vs. 'administrative duty' and show a definite impact of fire fighting tasks on mortality.

The professional fire fighters in Hamburg and mostly all over Germany are civil servants (German: 'Beamte'). This includes remarkable social benefits. Very few leave the department once they joined; almost all have lifelong careers in the fire departments. Hence, fire fighters over 55 years are still in active duty at the scenes. Hence the passion, when the political discussion to raise the retirement age to 65 years started.

The lower overall mortality does not indicate, however, that there were no other specific causes of death which increased the risk of death of fire fighters. A case-control study in the USA on on-duty deaths of active fire fighters has shown increased risks of death by coronary heart disease during fire suppression (OR = 64.1, 95% CI 7.4–556); training (OR = 7.6, 95% CI 1.8–31.3) and alarm response (OR = 5.6, 95% CI 1.1–28.8). The rate of on-duty deaths caused by coronary heart disease is reportedly higher than in other comparable occupational groups such as police or emergency services [8]. Other specific causes of deaths with higher than normal mortality in fire fighters are reported such as certain kinds of brain or colon cancer, leukemia, kidney and urethra cancer, prostate and bladder cancer [9-12].

### Mortality of early retirees

Our study confirms findings that showed an elevated SMR for persons who retire early. We observed a SMR of 1.35 (95% CI, 1.13–1.60) in reference to the general German population and a SMR of 1.71 (95% CI, 1.18–2.50) in the Cox-Regression in reference to all others. We did not observe any beneficial effect of early retirement as documented in a Danish study [13] and the Whitehall II study on effects of normal retirement. [14].

In a Danish population-based study the disability benefit recipients showed were markedly elevated mortality [15]. Retirement in itself seems to be a risk factor for early death. In a British study men who were unemployed had a RR of 2.13 (95% CI, 1.71–2.65, men who retired early for reasons other than illness had still a significantly higher mortality compared with employed men (RR 1.87, 95% CI, 1.35–2.60) [16].

A study of past employees of Shell Oil, USA, showed a significantly higher mortality of employees who retired early at 55 and who were still alive at 65 (n = 839) had a significantly higher mortality than those who retired at 65 (n = 900) (hazard ratio 1.37; 95% CI, 1.09–1.73). Mortality was significantly higher for subjects in the first ten years after retirement at 55 compared with those who continued working (1.89; 95% CI, 1.58–2.27). The significant difference, however, showed only after adjusting to sex, calendar year of entry to the study, and socioeconomic status. Retired employees in the low socioeconomic category had a higher mortality than retirees in the high category (1.17, 95% CI, 1.01–1.36) [17].

Results from the British Regional Heart Study indicated that men who retired early for reasons other than illness had a significantly increased risk of mortality compared with men who remained continuously employed (relative risk 1.87 (95% CI, 1.35–2.60)). [18] Early retirement was associated with higher mortality in a construction workers cohort in Germany (RR, 1.50; 95% CI, 1.20–1.88) [19].

### Reasons for retirement, reasons for non-fitness and possible risk factors for higher mortality of retirees

Our study was limited by the fact that we could not include common risk factors for elevated mortality such as cardiovascular risk factors (e.g. blood pressure, lipid profile [20]), psychosocial risk factors (e.g. stress, life event impact, traumatic experiences, depressive disorders [21-23]) or exposure factors to toxic gases (e.g. carbon monoxide). The social medicine department which handles the retirement cases of the City of Hamburg does neither publish nor hand out detailed statistics on reasons for retirement of public employees despite multiple requests.

However, we can presume that 'reasons for early retirement' were almost identical to 'reasons for restriction of fitness' because of medical problems during active duty. From studies of the occupational health service of the Fire Department we know that the reasons for restricted fitness because of medical problems, both temporary or permanent, were cardio-vascular diseases in 39% of all cases (N = 230) and 44% in fire fighters over 50 years (N = 132), musculoskeletal diseases (25% and. 21%), respiratory disorders (5% and 6%), injuries & surgeries (9% and 5%), metabolic disorders (3% and 5%) and psychiatric disorders including addiction and abuse (6% and 5%) [24]. High blood pressure accounted only for 5% of the medical fitness restrictions but was prevalent in 20% and 23% of the unfit fire fighters.

This pattern of diseases in events of non-fitness is distinctly different from reasons for retirement because of ill health in an analysis of retirees from the National Health Service, United Kingdom, which listed musculoskeletal (49%), psychiatric (20%), and cardiovascular conditions (11%) as most common reasons [25].

The rate of high blood pressure in Hamburg fire fighters is consistent with findings from other studies which reported a prevalence of high blood pressure between 20% and 23%, the majority of the men were untreated [26].

### Unspecified risk factors

Despite the pronounced healthy worker effect, our study yields several results of subgroup SMR and Cox-regression analyses which support the assumption that occupational hazards in fire fighting, which are not specified here such as stress, raised the mortality.

First, the SMR of fire fighters who worked more than 50% of their time in administrative units 0.53 (95% CI, 0.35–0.78) is lower than the mortality of persons who worked more than 50% in active fire fighting with 0.79 (95% CI, 0.74–0.84). Second, we observed a striking difference in mortality between rank groups. This confirms results of studies which show a reduced mortality in higher socioeconomic groups [27]. However, this difference could also reflect different tasks and job exposure profiles. Higher ranks are usually not part of the attack or rescue teams. Third, causes for early retirement are partly diseases often caused or triggered by the job. The elevated SMR for those persons may reflect individual susceptibility in combination with or reaction to special hazards from the job.

We are unable to forward any explanation for the rise of SMR in the group 5 to 10 years of duration of employment. As causes and circumstances of death could not be included in this study, a discussion of reasons for this finding was considered speculative by the authors.

The decreasing mortality with later date of entry is indicative of major changes in the work environment during the study period. Tactics and safety equipment for fire fighters were improved. Accident rates fell due to better techniques and safety equipment. Especially the widespread introduction of heavy respiratory protection equipment in the 80'ties lowered the exposure to fumes and gases drastically. In Hamburg respiratory protective gear was already used end of the 70'ties. [28] On the other hand, during the study period changes have occurred which may have influenced mortality negatively e.g. plastics were introduced *en mass *into the household environment and hence became part of structural fires resulting in toxic and carcinogenic burn products.

The multivariate analysis showed a considerably higher relative risk for fire fighters who joined the department after 30 years of age (SMR, 2.38). For this result we are also unable to provide any reasonable explanation as it is in contrast to findings of other studies [29]. In the absence of other explanations and due to the low numbers it could be a chance finding.

## Conclusion

In summary, we could confirm in our German study the results of previous studies of a lower-than-normal mortality of fire fighters. As expected, a strong healthy worker effect with all its components was observed. The findings also suggest that the intensive medical surveillance is beneficial to the overall health and mortality of professional fire fighters.

However, results indicate negative effects of type of task, rank status and early retirement on mortality. It appears that fire fighting and rescue services have a distinct negative influence on mortality.

## Abbreviations

SMR, Standardized Mortality Ratio

CI, confidence interval

RR, relative risk

HWE, healthy worker effect

SCBA, self contained breathing apparatus

NFPA, National Fire Protection Association/USA

Stress-ECG, stress-electrocardiogram

HFD, Hamburg fire department

## Competing interests

The study was financially supported by the Fire Department of the City of Hamburg accounting for about 25% of the budget. None of the authors received personal remuneration from this fund. No other competing interest to declare.

## Authors' contributions

NLW initiated and coordinated the study and participated together with DFJ, MP and JB in its design and result interpretation. PK and DFJ were responsible for gathering the data, data entry and quality control. Together with AK they carried out the statistical analysis. NLW, DFJ and PK wrote the manuscript. TO provided information on reasons of non-fitness and helped revise the discussion. All authors read and approved the final version.
